# Twister3: a simple and fast microwire twister

**DOI:** 10.1088/1741-2552/ab77fa

**Published:** 2020-05-19

**Authors:** Jonathan P Newman, Jakob Voigts, Maxim Borius, Mattias Karlsson, Mark T Harnett, Matthew A Wilson

**Affiliations:** 1 Department of Brain and Cognitive Sciences, MIT, Cambridge, MA, United States of America; 2 Picower Institute for Learning and Memory, MIT, Cambridge, MA, United States of America; 3 McGovern Institute for Brain Research, MIT, Cambridge, MA, United States of America; 4 SpikeGadgets LLC, San Francisco, CA, United States of America; 5 Open Ephys Inc, Cambridge, MA, United States of America

**Keywords:** tetrode, microwire, freely-moving, extracellular electrophysiology, open-source, neural recording

## Abstract

**Objective.:**

Twisted wire probes (TWPs, e.g. stereotrodes and tetrodes) provide a cheap and reliable method for obtaining high quality, multiple single-unit neural recordings in freely moving animals. Despite their ubiquity, TWPs are constructed using a tedious procedure consisting of manually folding, turning, and fusing microwire. This imposes a significant labor burden on research personnel who use TWPs in their experiments.

**Approach.:**

To address this issue, we created Twister3, an open-source microwire twisting machine. This machine features a quick-draw wire feeder that eliminates manual wire folding, an auto-aligning motor attachment mechanism which results in consistently straight probes, and a high speed motor for rapid probe turning.

**Main results.:**

Twister3 greatly increases the speed and repeatability of constructing twisted microwire probes compared to existing options. Users with less than one hour of experience using the device were able to make ~70 tetrodes per hour, on average. It is cheap, well documented, and all associated designs and source code are open-source.

**Significance.:**

Twister3 significantly reduces the labor burden of creating high-quality TWPs so electrophysiologists can spend more of their time performing recordings rather than making probes. Therefore, this device is of interest to any lab performing TWP neural recordings, for example, using microdrives.

## 1. Introduction

Since their introduction [1], twisted wire probes (TWPs; e.g stereotrodes [1] and tetrodes [2, 3]) have been a reliable method for obtaining single-unit extracellular spiking data in freely moving animals. They are cheap (~5–10 USD/m using Sandvik PX000004), small enough to cause minimal inflammation, and sufficiently biocompatible to be used over many months [4–6]. Their contacts are close (~10–20 *μ*m) and therefore allow much improved unit separability compared to single wire probes [7].

Compared to other commonly used electrode types, they are mechanically flexible, such that they move with neural tissue, rather than behaving like a rigid, skull-coupled beam. This improves long term stability compared to, e.g. silicon probes^1^, which require second-to-second removal of artifacts due to relative shear motion (‘drift’) in order to obtain classifiable action potential waveforms [8–10]. In contrast, TWPs allow units to be tracked continuously over multi-day periods with minutes-long time-scale drift correction [4], which is less deleterious to unit classification than fast drift [10]. Although the introduction of modern silicon [11, 12] and carbon-fiber probes [13] offer major advances in terms of channel density and size, respectively, and polyimide-substrate and mesh-electronics probes provide orders of magnitude decreases in mechanical stiffness [14], TWPs will remain a ubiquitous recording method for the foreseeable future due to their simplicity, ease of implantation, good performance, and low cost.

Although simple to make [15], constructing TWPs is a tedious and time-consuming process. For modern, easy-to-assemble microdrive designs [5], making TWPs is a rate-limiting step. Typically, TWPs are made in three actions [15]. First insulated tungsten or nickel-chrome (‘nichrome’) resistance wire is drawn from a spool and folded by hand 1 or 2 times to make stereo- or tetrodes, respectively. Next, the folded wire is draped over a smooth metal rod. The free end of the wire bundle is loosely coupled to a motor armature using a mechanical^2^ or magnetic^3^ mechanism. The motor then twists the wire bundle into a helix. Finally, a hot air gun is used to melt and fuse the insulation on the wire helix, forming a springy, implantable probe.

**Figure 1.**
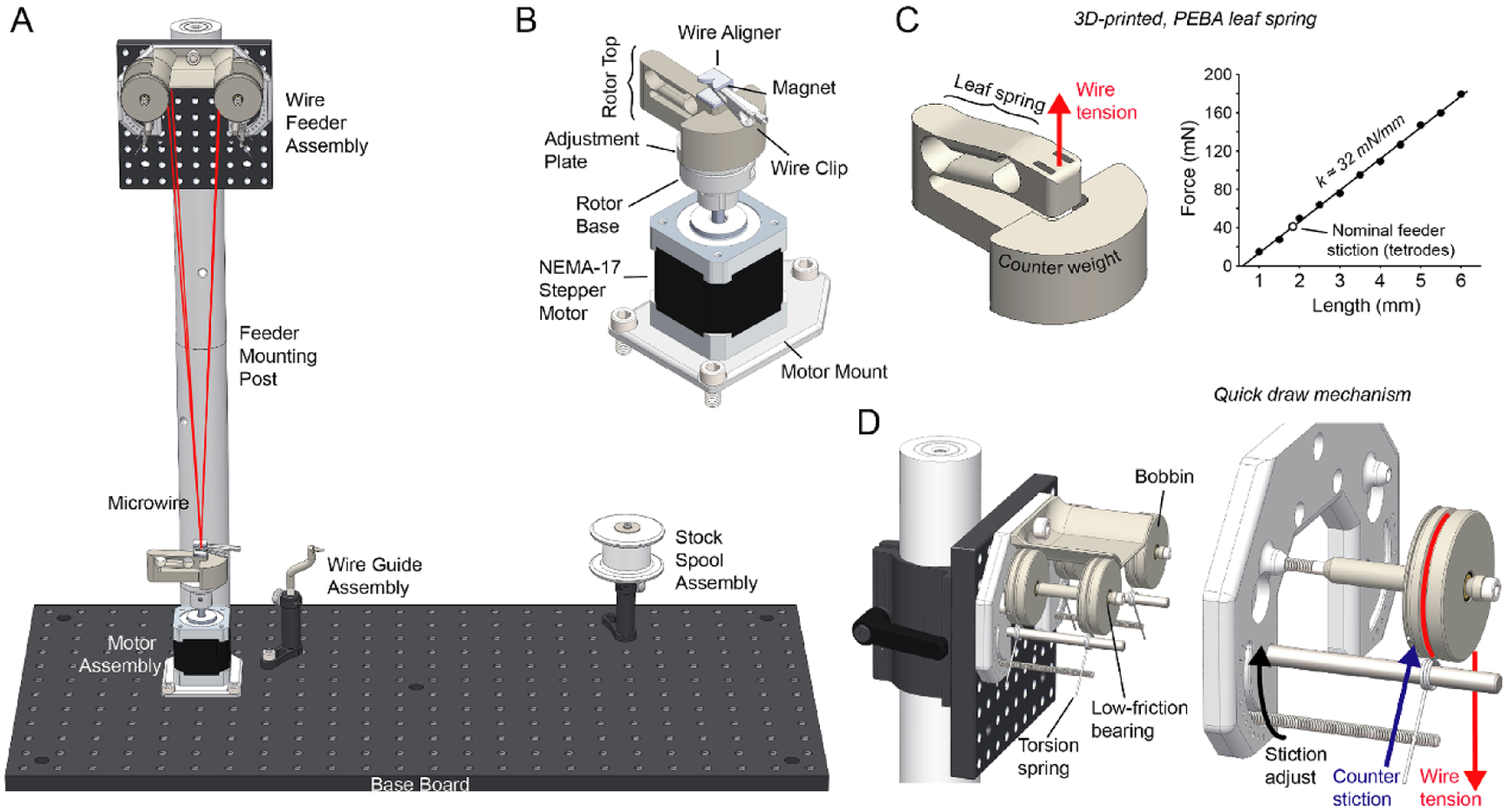
(A) Overview of Twister3’s mechanical components. The motor and wire feeder assemblies are used to rapidly construct TWPs by drawing a wire bundle from the feeder, clipping it to the motor, and performing a twist. Additionally, the motor, wire guide, and stock spool assemblies are used to load wire onto the bobbins in the feeder after they are depleted. (B) Motor assembly. A NEMA-17 stepper motor is used to twist TWPs and reload bobbins. The wire clip, alignment jig, and magnet allow the wire bundle to be rapidly and reliably linked to the motor. The rotor base and adjustment plate allow one-time adjustment to achieve perfect alignment between the wire bundle and the motor axis. (C) *(left)* The 3D printed leaf spring showing deformation under tension. The shape of the spring permits approximately vertical deformation of the wire attachment point so that the center axis is maintained as the bundle is shorted due to twisting. *(right)* Spring tension as a function of vertical deformation. Best fit line indicates a spring constant of 32 nM mm^−1^. The white dot indicates the spring deformation needed to oppose the wire-feeder’s stiction setting for TWPs made in our lab. (D) *(left)* Wire quick draw mechanism. *(right)* Isolated single bobbin indicating the wire tension, due to the leaf spring in (C), and counter stiction due to the adjustable torsional spring.

Recently, SpikeGadgets^4^ introduced a tetrode twisting machine that provides an arrangement of four pre-wound wire bobbins that allows the user to draw a multi-wire bundle without the folding step^5^. Because folding is the most time consuming part of the TWP making process, this device greatly improves the speed at which TWPs can be created. Additionally, this method minimizes human wire handling, which is beneficial because wire ends up in direct contact with neural tissue and is difficult to clean. Despite these advantages, the high-price of its custom-designed components (~10 000 USD) and complexity of this device has hampered its adoption. Inspired by the SpikeGadgets design, we collaborated to create an open-source twisting machine that is approximately ten times cheaper and ten times faster (figure 1(A)). Our device uses a high-speed stepping motor and modern micro-stepping driver to increase twisting speed while maintaining precise control of motor acceleration and smooth motor actuation. A new counter-balanced, auto-aligning spring system provides constant tension on wire during twisting. Our design allows a moderately trained (~1 hr of experience with device) operator to make ~70 TWPs per hour.

Here we present descriptions of how this device works, materials and assembly information, and electronics designs. We provide detailed usage instructions, an exploration of probe mechanics with respect to twisting parameters, TWP construction time measurements, and show data obtained with tetrodes made using this machine. All designs and source code associated with this project can be found on the Twister3 git repository^6^.

## 2. Notable design elements

### 2.1. 3D-printed leaf spring for fast twisting

In order to achieve straight TWPs, wire must be twisted together while under tension. In all other existing designs, a weight is hung from the wire bundle and a motor is loosely coupled to the weight in a way that does not constrain axial motion. This provides constant tension on the wire bundle due to gravity while allowing the helix to decrease in length as it is twisted. A variant of this method uses magnets hold the weight in place^7^, but, in our experience, this is unnecessary and is prone to causing wire breakage due to the nonlinear force/distance relationship of magnetic attraction.

A key design criterion for our device is that TWPs need to be turned very quickly. We aimed for < 10 seconds of twisting time (time when motor is in motion) per TWP. Assuming 100 total revolutions (upper estimate for tetrodes) this translates to an average turn rate of 600 RPM. Because previous methods rely on loose motor coupling, they were unsuitable to meet our speed requirements. For example, the SpikeGadgets machine, original Open Ephys Twister, and Neuralynx Spinner-2.0 are limited to approximately 60 RPM. The high centripetal forces involved in rapid turning inevitably leads to instability of the coupling mechanism, causing the bundle to vibrate wildly. Therefore, we sought to rigidly constrain the motor and wire bundle in the turning plane, but still maintain freedom in the axial direction to allow bundle tensioning and shortening during the turning process. To meet this goal, we made use of selective laser-sintered polyether block amide (PEBA) to create a monolithic, combined leaf spring/wire-retention mechanism (figure 1(B)). PEBA provides rubber-like mechanical qualities resulting in a spring-constant low enough for use with microwire (~32 mN mm^−1^ in the relevant range of motion (figure 1(C)).

### 2.2. Auto-aligning wire/motor interface

To interface the microwire bundle with the motor, the bundle is clipped together using a standard, ferrous alligator clip that has been coated in insulating shrink wrap. The bundle is then drawn towards the motor while the leaf spring is lifted with with the user’s free hand. While the leaf spring is under tension, the alligator clip is attached to a strong neodymium magnet on the leaf spring assembly, which provides the bundle-to-motor linkage (figures 1(A), (B)). During this process, a 3D-printed alignment jig on top of the magnet automatically guides the microwire bundle in line with the motor’s axis of rotation. This alignment jig must be made from a material that can withstand the high temperature of the hot air during the fusing process and is smooth enough to ensure it does not snag the microwire as it guides it into position. We have found that 3D-printed rhodium-plated brass works very well for this purpose. The leaf spring is then slowly lowered until it reaches equilibrium with upward wire tension. At this point the motor can be turned.

Because of print tolerances and the fact that the leaf spring does not deform exactly vertically, the resting position of wire bundle will likely not be perfectly co-linear with the motor axle. To account for this, the motor mount consists of a two pieces: the base, which is rigidly concentric with the motor shaft, and an alignment plate. The alignment plate is friction fitted within the base and provides ~2 mm of omnidirectional planar adjustment (figure 1(B)). This plate should be moved until the resting position of the wire bundle is in line with the motor axis.

This wire attachment mechanism has two advantages over existing designs. First it can be used rapidly because of magnetic coupling. Second, because the microwire bundle is automatically aligned with the motor’s axis of rotation, high-turn rates do not result in oscillations or instability. We have found that this is a critical feature in order to produce straight, even-pitch, and consistent TWPs using very fast turn speeds.

### 2.3. Quick-draw wire feeder

To further increase the speed at which TWPs can be made, microwire needs to be drawn and attached to the motor quickly. Ideally, this process should occur with as few separate actions being taken by the operator as possible. To facilitate the rapid draw of wire from stock spools, we designed a torsion spring-based feeding assembly that allows wire to be quickly drawn from stock feeder bobbins (figure 1(C)). This mechanism applies enough friction to feeder bobbins to counter increased wire tension during a twist, transferring all slack compensation to the leaf spring (figure 1(B)). The holding (stiction) force of this mechanism is adjustable to account for the elastic deformation of different wire materials. We have found that, when working with standard tetrode wire^8^, the 2nd stiction setting (corresponding to a threshold of ~11.5 N per spool), is adequate to counter wire tension and therefore prevent spools from improperly feeding during a twist. However, the proper stiction setting is dependent on the microwire material and will need to be adjusted for other wire types.

### 2.4. Motor control hardware for smooth turning

**Figure 2.**
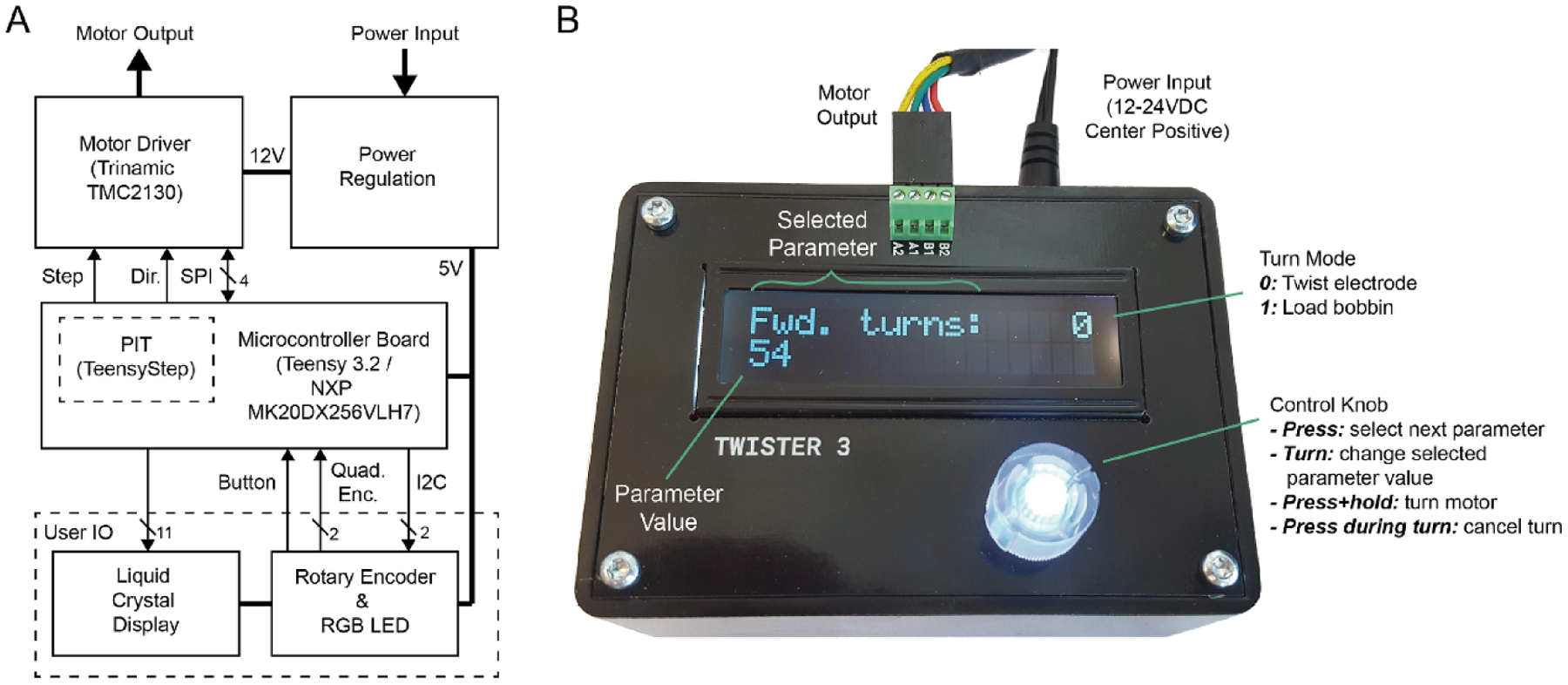
(A) Control electronics block diagram. All user IO is provided via a combined rotary encoder and button. The Teensy’s NXP MK20DX256VLH7 microcontroller provides a programmable interrupt timer (PIT) to control step commands to the motor driver, independent of nominal operation. (B) Control box with callouts showing features, connections, and controls.

To obtain precise control over motor acceleration, speed, and position we to used a bipolar stepper motor to perform wire twisting. We have found that, due to their discretized motion, stepper motors can vibrate resonantly with taut microwire, resulting in irregular twists and wire damage. To overcome this issue, we drive our motor using an advanced microstepping driver (figure 2(A)); Trinamic TMC2130). In our case, we use 200 steps/revolution (1.8°) motor. Microstep commands from the microcontroller are provided at 16 microsteps/step, which are further interpolated to 256 microsteps/step by internal driver circuitry. This results in a motor update resolution of 3 200 microsteps/revolution (0.113°), and a motion discretization of 51 200 steps/revolution (0.007°). Therefore, the motor operates approximately as smoothly as a continuous DC motor but with much improved motion control dynamics.

We used an Arduino-compatible Teensy 3.2^9^ microcontroller module to perform step timing calculation. Its NXP MK20DX256VLH7 Cortex-M4 includes an integrated programmable interrupt timer (PIT) which is used to provide jitter-free step commands to the motor driver while acceleration calculations are performed^10^.

## 3. Usage

The following sections provided detailed instructions for using Twister3 to load wire and make TWPs. This content is aided by an instructional video available on YouTube (appendix A).

### 3.1. Using the control box

The control box (figure 2(B)) is powered using a 12 V DC center-positive barrel jack that supplies at least 1.5A. It has a single user input: a control knob consisting of a combined quadrature rotary encoder and tactile push-button. This knob permits the following user actions:

- **Press:** cycle through different settings (forward turns, backward turns, turn speed, turning mode)
- **Turn:** increment or decrement the selected setting depending on turn direction.
- **Press and hold:** execute the turn sequence using the current settings
- **Press during motion:** cancel the twist and stop the motor immediately.

The control box is used to perform two tasks: twisting electrodes (mode 0) and loading bobbins with microwire (mode 1). The turn mode is selected and changed using the dial on the controller. The selected turn mode is shown in the upper right corner of the liquid crystal display (LCD). After a mode is selected, all turning parameters (speed, forward and backwards turns) pertain to that mode only. All parameters are stored in non-volatile memory when a turn is started by pressing and holding the control knob. In the following sections, we detail how to use the mechanical components for making TWPs and loading bobbins with stock wire.

### 3.2. Loading bobbins

**Figure 3.**
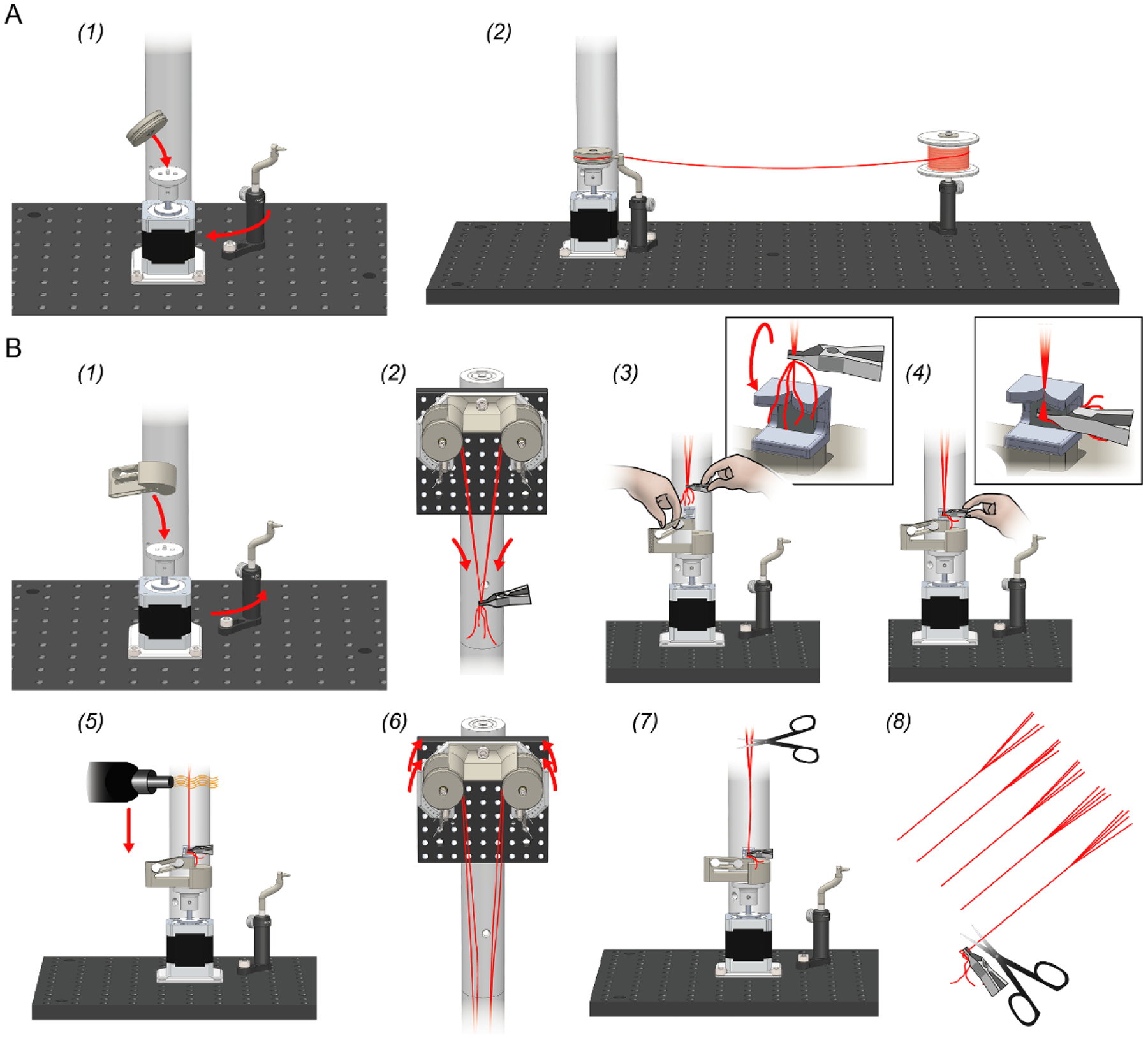
Bobbin loading and TWP creation procedures. (A) Summary of the bobbin loading procedure. See text for details. (1) The leaf spring is removed and a bobbin is magnetically mated with the rotor. The wire guide is moved into close proximity to the wire groove on the bobbin. (2) Wire from the stock spool is threaded through the wire guide and around the bobbin. The control box is then used to load a desired amount of stock wire. (B) Summary of the TWP construction procedure. See text for details. (1) The leaf spring is attached to the base rotor and the wire guide is moved out of the way. (2) The loose wire is alligator clipped and drawn from the bobbins. (3) The user’s free hand is used pull up on the twisting attachment’s leaf spring until under slight tension. (4) The alligator clip is drawn down and flipped 180° to meet the magnet within the alignment jig (insets). This procedure improves the alligator clip’s grip on the bundle. A twist can then be performed. (5) After the twist, wires are fused *starting from point at which they separate towards the bobbins* using a hot air gun. (6) The bobbins are simultaneously rolled forward forward to release tension on the wire. (7) The loose wire is cut e above the point at which the wires are fused, and the alligator clip is removed from the magnet with a finished TWP. (8) The finished TWP is cut into a storage box.

Before twisting electrodes, the bobbins on the wire feeder assembly must be loaded with microwire (figure 3(A)). The following paragraphs detail the bobbin loading procedure. A video of the bobbin loading process is available to aid these instructions (appendix A).

First, the bobbins must be removed from the wire feeder assembly. To do this, the wire shield is removed by unscrewing its M6-retention screw as indicated in figure 6(6). Next, the bobbins are removed by unscrewing the M3 bolt that serves as the bobbin axle (figure 6(4)). The bobbins are then slipped off of each axle. If the bobbins have any left-over microwire, it should be removed and the wire grooves should be examined in to ensure they are clean of dirt and debris. They can be wiped with a cotton swab and water if they require cleaning.

Next, the 3D-printed leaf spring is removed from the rotor base. Because each bobbin has embedded magnets that mate with those on the rotor base, after the leaf-spring is removed, a bobbin can be placed onto to rotor base (figure 3(A1)). Once the bobbin is in place and reasonably centered using the adjustment plate (figure 8(5)), the wire guide should be positioned such that its tip points directly into the center of the wire groove on the bobbin. Properly loaded bobbins will have microwire tightly wound around their center groove. This relies on carefully adjusting the position of the wire guide such that it is tip is located about 1–2 mm from the center groove (figure 3(A1)). After this, wire is feed from from the stock spool through the wire guide and wrapped once around the bobbin in its center groove (figure 3(A2)).

Finally, to load wire onto the bobbin, the controller is set to mode 1 (figure 2(B)) and then the desired loading speed and number of revolutions are selected. We have found that 100 RPM works well for most wire (figure 2(B)). The circumference of the bobbin is ~10 cm. Therefore, the length of wire loaded on the bobbin is *turns* × 10 *cm*. Including wastage, this results in a conservative estimate of 1 TWP per turn (table 1). After the desired parameters have been entered, loading is started by pressing and holding the controller button. *Be careful not touch moving parts during this process*: the microwire needs to have constant tension to ensure it is properly loaded on the bobbin. The loading processes should be monitored as it begins to ensure that microwire is being accepted by the bobbin. If there is an issue, pressing the knob on the control unit will halt the process so it can be corrected.

Once loading has completed, the process is repeated for the for the remaining bobbins. After each bobbin has be loaded with microwire, they are put back on their axle on the wire feeder assembly. Loose wire ends should point inward on both sides of the assembly (figure 3(B2)). Finally, the wire shield is replaced (figure 6(6)).

### 3.3. Making twisted wire probes

The following steps detail TWP construction using Twister3. If you are interested in making stereotrodes instead of tetrodes, follow the same steps but only use two bobbins on diagonally opposing sides of the feeder. A video of the TWP construction process is available to aid these instructions (appendix A).

If it is not already in place, the leaf spring must be attached to the base rotor using its magnetic interface (figure 3(B1)). With the leaf spring in position, wire from each of the bobbins is grouped using a finger pitch. The bundle is then clamped using the alligator clip (figures 3(B2), 7(B)). The wire bundle does not need to be in a certain position within the clip or be tightly grouped Additionally, each wire does not need to begin at exactly the same length from the feeder because those with more slack will not pull on their bobbin until they it is equal in length to the shortest wire. At the point that all wires are drawing slack, they will be equidistant from the wire feeder.

Once the wires are clamped in the alligator clip, it is flipped 180° such that the wire bundle wraps around the bottom of the clip and exits its rear face (figure 3((B3), inset)). This will ensure that the wire does not slip out of the clip as it is draw from the feeder. Do not worry if the wire is not tightly bunched as the twist alignment jig will keep the bundle exactly concentric with the axis of rotation during twisting. Using your free hand, the twisting attachment’s leaf spring is lifted until under slight tension, about 1.5 cm (figure 3(B3)). Then, with the leaf-spring raised, the alligator clip is drawn down to meet the magnet on the twisting attachment, feeding the bundle into the aignment jig (figure 3(B4)). After the clip is magnetically mated, the leaf spring is smoothly lowered until it is in equilibrium with the upward force produced by the wire. Each of the wires should be pulled straight. Do not let the spring snap back under its tension, as this will leave slack in the wires. If any wire has slack, its bobbin can be turned backwards slightly until it is taut. Finally, ensure all wires are guided through the center of the alignment jig (figure 3((B4), inset)) and the loose ends are not interfering with the taught portion of the wire. If so, they should be cut before performing a twist.

Before performing the first twist in a session, ensure that the alignment plate has been adjusted so that the point at which the wire exits the alignment jig is co-axial with the motor shaft (figure 8(5)). To do this, adjust the position of the plate until until the point at which the wires enter the alignment jig entry point does not ‘wobble’ with respect to a steady background object when the motor is manually turned back and forth. This should only need to be done once per session. After alignment, the controller is set to mode 0 and the desired number of turns and turn speed are selected (figure 2(B)). We use 900 RRM for our wire (table 1). These settings only need to be entered once because they are saved to non-volatile memory by the controller every time they are changed. The button is then pressed and held to start a twist.

After the twist is complete, the wires are fused using hot air. The exact parameters of the hot air gun will dependent on the melting point of microwire insulation material. For polyimide, we have found that 480 ~ 480°C is a good temperature. We have found that the air flow is not critical, but should not be so high that it deforms the taught wire during the fusing process. Settings for our hot air gun (table B3) lab are provided in table 1.

Once the air gun has reached a stable temperature, the nozzle is held ~5 mm from the from the point at which the twisted microwires separate towards the feeder bobbins (figure 3(B5)). It is then moved downward slowly and smoothly (~2 cm s^−1^) until it reaches the wire alignment jig. The direction is then reversed and it is raised until the nozzle returns to its initial position at the wire separation point. The nozzle is then moved away from the wire. Starting from the top and fusing downward is important: we have found that fusing from the bottom and moving upwards will cause the lower portion of the TWP to ‘absorb’ slack from above resulting in a very fine twist pitch and a TWP that is shorter than intended.

After wire fusing, both hands are used to simultaneously roll each of the bobbins forward until the leaf spring relaxes and there is no tension on the microwire (figure 3(B6)). The loose wire above the fused TWP is then cut using scissors (figure 3(B7)). When performing this cut, make sure to leave enough free wire for connectorization. Finally, the alligator clip is removed from the magnet in the wire alignment jig and the finished TWP is cut into a storage box (figure 3(B8)). The process can then be repeated for the next TWP.

### 3.4. Selecting device parameters

Choosing twisting parameters will require some experimentation in order to produce TWPs with the desired geometric and mechanical properties given the user’s choice of wire, implant type, and animal model. In our labs, we use Twister3 to make tetrodes for microdrive implants in both mice and rats. The operation settings that we use are shown in table 1.

**Figure 4.**
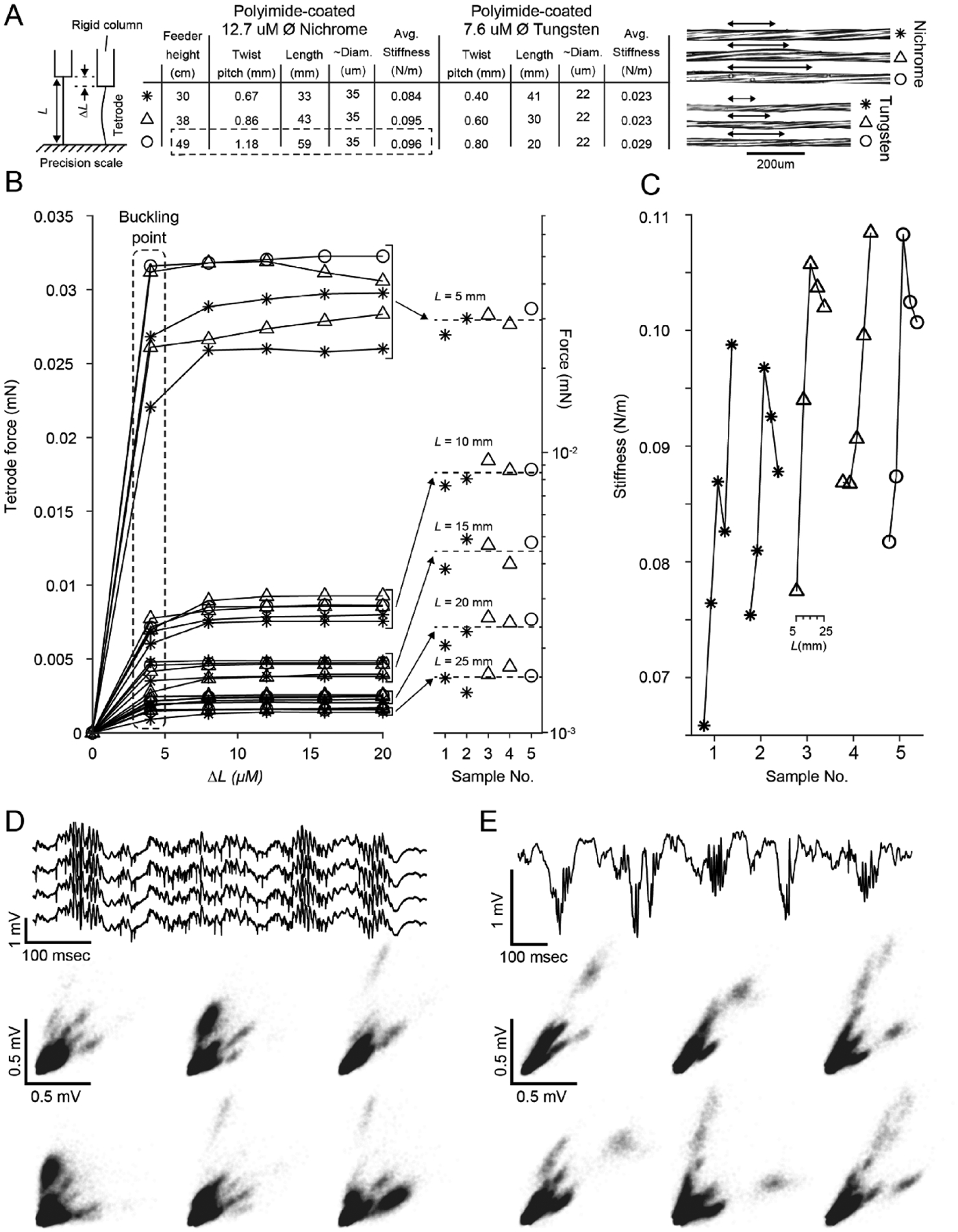
Tetrode characteristics for different twist pitches and wire types. (A) *(right)* Diagram of the mechanical test. Tetrodes were attached to a rigid column and the exposed portion cut to a length *L*. The rigid column was lowered in small increments using a micro-manipulator onto a precision scale and the resulting force was measured to find the buckling point. *(middle)* Feeder height versus twist pitch, probe length, diameter, and average stiffness across exposed lengths when using nichrome or tungsten microwire of different diameters. Aside from the feeder height, all other construction parameters were kept the same as the rat column of table 1. The circled row indicates the parameters used to create the tetrodes that produced the data in panels (D) and (E). *(right)* Micrographs of sections of probe shanks for each twist pitch and material presented in the table. Each arrow-line is a quarter pitch. (B) *(left)* Compressive force versus depth lowered (*δL*) onto a rigid surface. Each line is a single tetrode sample cut to one of 5 exposed lengths (*L* = 25, 20, 15, 10, and 5 mm). Data point symbols correspond to the nichrome portion of table in (A) for various probe lengths. The buckling force (value at which there is no increase in restorative force with *δL*) is length dependent. Different exposed probe lengths form clear groupings with the bucking force increasing as the probe length gets shorter. *(right)* Buckling forces for each sample on a log scale. The buckling force is clearly grouped for each probe length but is not affected by twist pitch. (C) Stiffness versus probe length for each of the nichrome samples tested in (A). Of the parameters tested, longer, wide-pitch TWPs tended to be stiffest. (D) Example hippocampal mouse CA1 recording during sleep. (top) Raw voltage trace from a single tetrode. Three sharp wave-ripple events are clearly visible in the trace. *(bottom)* Spike amplitudes for each combination of two wires on the tetrode. (E) Example hippocampal rat CA1 recording during sleep. *(top)* Raw voltage trace from a single electrode. Multiple sharp wave-ripple events are clearly visible in the trace. *(bottom)* Spike amplitudes for each combination of two wires on the tetrode. Recordings in (D) and (E) are skull referenced.

Two settings of note are the height of the wire feeder above the motor assembly, which determines the probe length and microwire twist pitch and the bobbin stiction threshold which determines the wire tension during twisting and drawing. Figure 4 shows the effect of changing the wire feeder height on resulting tetrode characteristics for 12.7 *μ*m diameter, polyimide coated nichrome and 0.6 *μ*m, polyimide coated nichrome mircowire types. For both wire types, lowering the feeder closer to the motor, and therefore increasing the angle of wire divergence from the axis of rotation does decrease twist pitch and probe length for a given number of turns. However, the buckling point (figure 4) and stiffness (figure 4) of tetrodes across feeder heights for each material are remarkably stable. Probe stiffness across materials and wire diameters is very different, as expected.

To test probe functionality, we produced tetrodes using the device settings in table 1. Using procedures that were approved by the Committee on Animal Care of Massachusetts Institute of Technology and followed the ethical guidelines of the US National Institutes of Health, these tetrodes were gold plated [15] and used in combination with microdrive assemblies [5, 16] to obtain recordings in the pyramidal cell layer of CA1 of mice and rats. As expected, these tetrodes reliably produced characteristic LFP and multiple, well-isolated units in both mice (figure 4(D)) and rats (figure 4(E)).

### 3.5. TWP construction time

**Figure 5.**
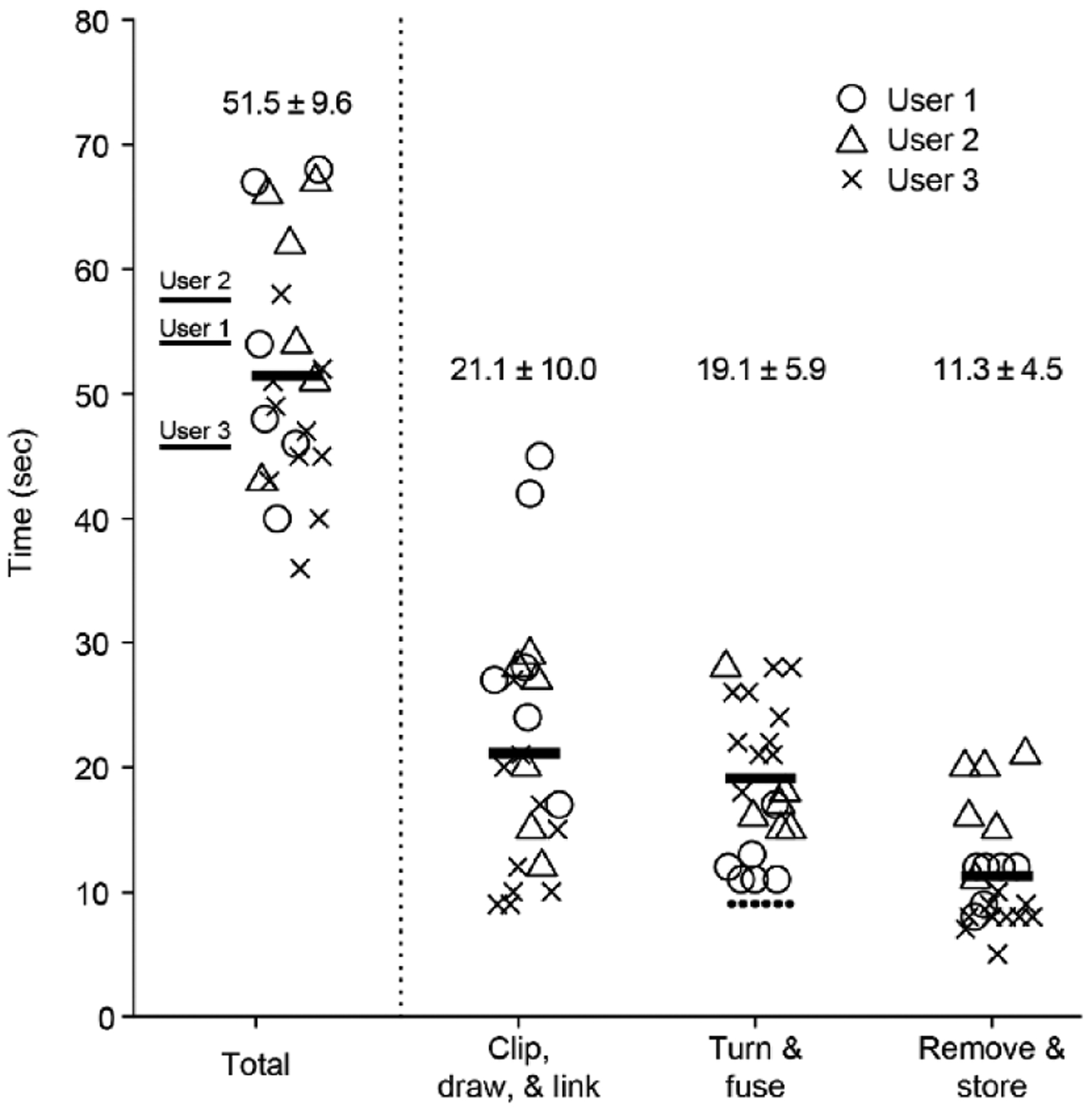
Tetrode construction time of users with ~1 hour of experience with Twister3. *(left)* Total construction time for each tetrode (symbols), average time across tetrodes for each user (thin lines), and across all tetrodes and users (thick line). *(right)* Timing for each step of tetrode construction. The dotted line above the turn and fuse step is the constant motor-in-motion time. Twister3 parameters were the same as the ‘Mouse’ column of table 1.

To quantify Twister3’s speed of operation, we measured the tetrode construction time of three users. All users had ~1 hour of experience with the device at the time of testing (figure 5). We divided speed measurements into three steps: *(1)* wire clipping/drawing, *(2)* wire twisting/fusing, and *(3)* tetrode removal and storage. Time trials were performed using the device parameters shown the ‘Mouse’ column of table 1. The motor-in-motion time was constant (9.1 s at 1000 RPM max speed) and therefore incurred a constant offset on step (2), as indicated by the dashed line in figure 5. All users were able to create tetrodes at a pace exceeding 1 tetrode/minute. However, each user had slightly different strategies when using the device. For instance, user 3, who has large amounts of experience with tasks requiring fine motor skills, was relatively quick for steps (1) and (3) and was relatively slow for the fusing step (2). User 1 had occasional difficulty clipping and the wire during step (1), increase their average time. These discrepancies indicate that there is room for future improvement and automation, especially with respect to wire clipping and fusing.

## 4. Materials and assembly

A potentially updated bill of materials (BOM) for electrical, mechanical and 3D-printed parts is available on a Google sheet^11^. Instructions for component assembly are provided in the following sections. For ease of reference and permanence, we provide snapshots of current BOMs in appendix B. However, the online versions should be used during Twister3 assembly to ensure up to date suppliers and error corrections.

### 4.1. Mechanical components

**Figure 6.**
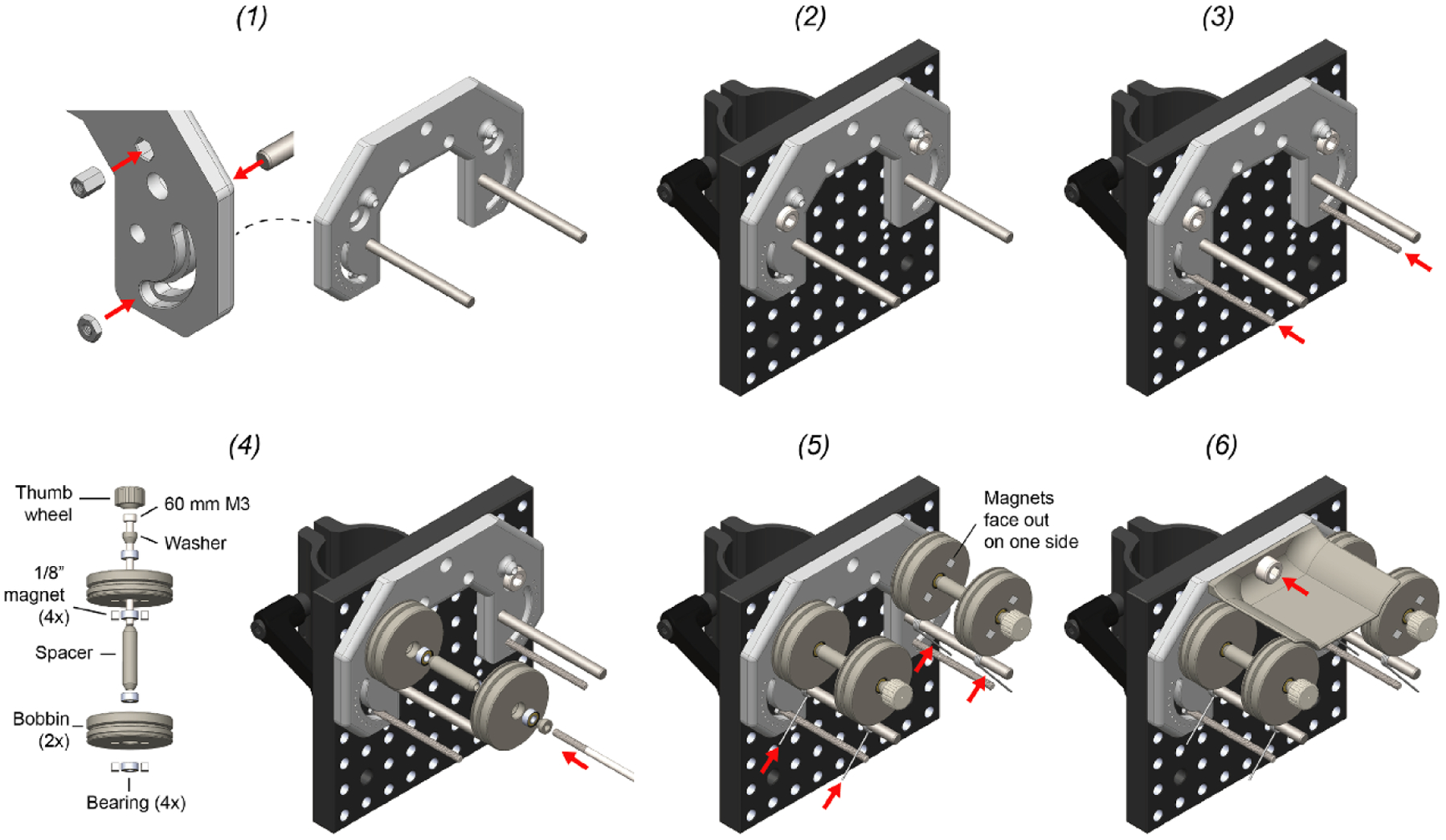
Wire feeder assembly. (1) Insert press-in components into the feeder base. This includes M3 nut (2x), M3 standoff (2x), and 3/16” diameter dowel pin (2x). This requires a mallet. (2) Mount the feeder base unto the C1545/M mounting clamp using M6 screws (2x). The top of the feeder should be flush with the mounting clamp. (3) Cut two 5 cm sections from the M3 threaded rod. Turn each section into the M3 nut which behind the feeder base. The position of the rod determines the stiction on each bobbin during wire draw. Lower positions provide less stiction. We have found that the second notch is a good position to start with. (4) Use the 60 mm M3 screw to mount the bobbin assembly to the standoff captive within the feeder base. Repeat for both sides. The thumb-screw head should be glued onto the M3 screw using epoxy prior to this step. (5) Thread a torsional spring onto the dowel pin. Squeeze it together and then set it between the threaded rod on one side and the shallow groove in each bobbin on the other. Repeat for each bobbin. (6) Install the wire shield above the bobbins using a single M6 screw.

**Figure 7.**
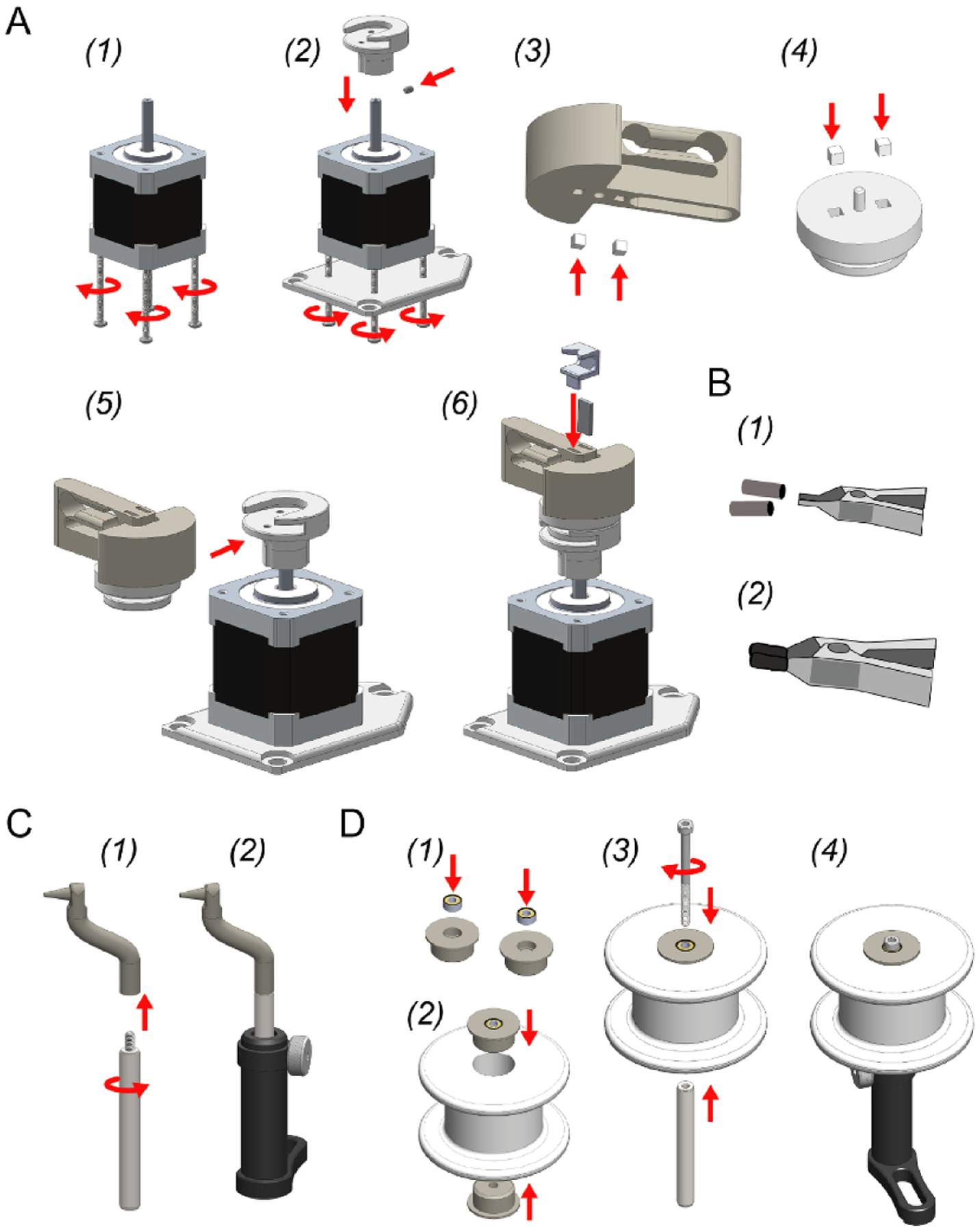
Other mechanical assemblies. (A) Motor assembly. (1) Remove the long, M3 step screws from the bottom of the stepper motor (4x). (2) Use 40 mm M3 screws (4x) to attach the motor mount to the bottom of the motor. (3) Fix the rotor base onto the shaft with the M3 set screw. (4) Press fit two magnets into the alignment plate *in the same orientation*. Make sure they are pushed down until recessed below the plastic surface so that they do not interfere with the flat mating surface of the piece. (5) Insert the alignment plate into the slot on the rotor base. (6) Attach two *additional* magnets on top of those you just inserted into alignment plate. Press the spring rotor onto these magnets to press fit them into the spring rotor base. This procedure ensures that magnets will be press fit into the spring rotor base with the correct polarity. Make sure the magnets are recessed below the bottom surface of the spring rotor so that it rests flat on top of the alignment plate. (7) Push the clip magnet into one of the slots on the spring rotor top. In the remaining slot, press the twist alignment jig into position over the magnet. (B) Wire clip assembly. (1) Put two pieces of heat shrink tubing over the wire clip jaws. (2) Shrink into position using the hot air gun. This prevents electrode wire from slipping during a draw. The clip can then be stuck under the wire alignment jig (figure 3). (C) Wire guide assembly. (1) Screw the wire-guide into a mini, 6 mm diameter optical post. (2) Push the optical post into a swivel post holder. (D) Stock spool assembly. (1) Push a bearing into each of the stock spool bearing cases. (2) Push each of the bearing cases into the stock spool of microwire. (3) Push a 40 mm, M3 screw through the two bearings and screw into a mini, 6 mm diameter optical post. (4) Push the optical post into a swivel post holder.

**Figure 8.**
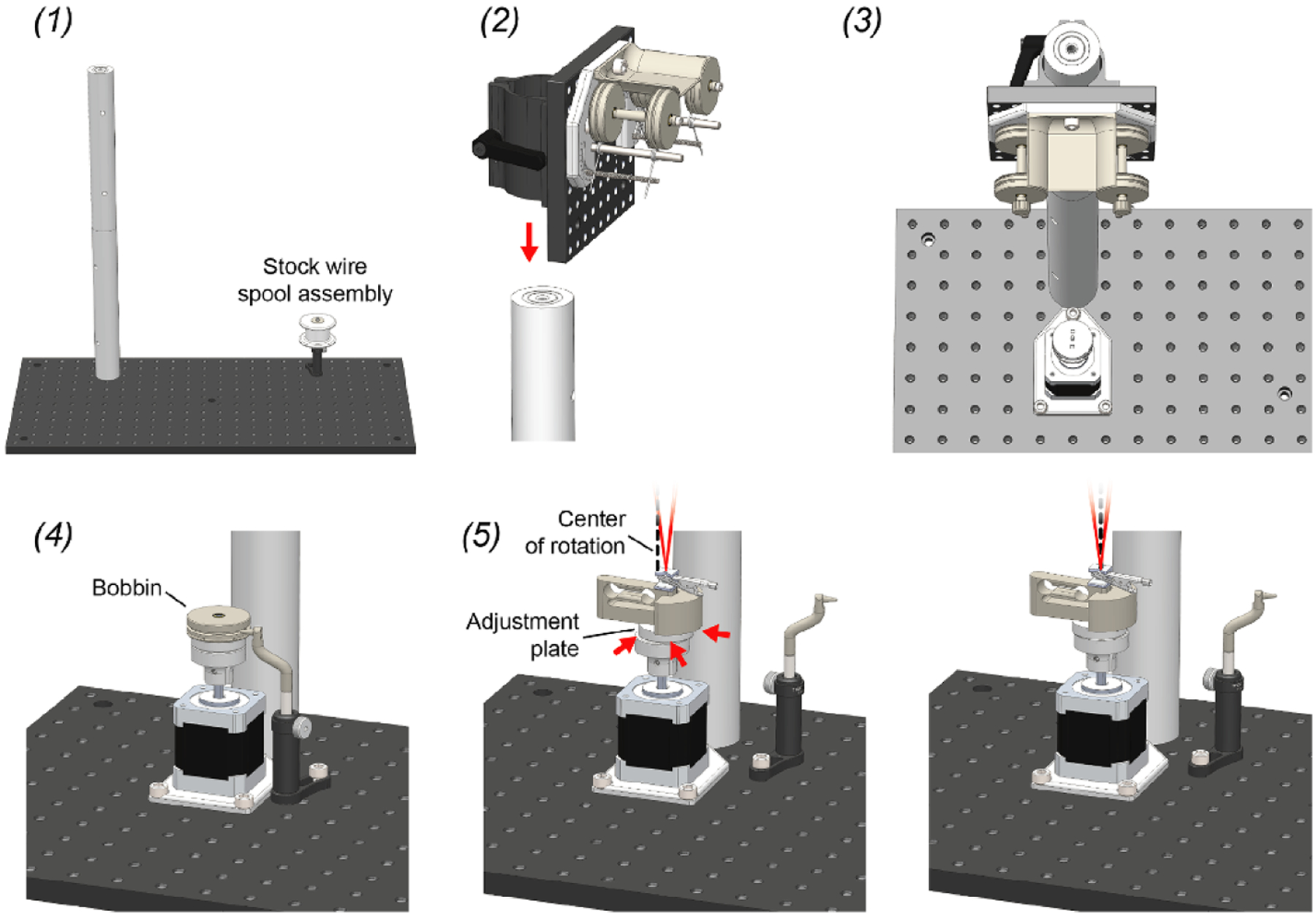
Twister3 assembly. 1. Screw together the large, 1.5” diameter mounting posts and then screw this long post into either the left or right side of the optical bread board. Mount the stock spool assembly in on the opposite side of the optical breadboard using a M6 screw. Its exact position does not matter. 2. Mount the feeder assembly on the post using the post mounting clamp on its back. 3. Mount the rotor assembly directly in front of the post, as close as it will go, using 3 M6 screws. 4. Mount the wire guide assembly into a position that is in close proximity to the motor assembly using a single M6 screw. The tip of the wire guide should be able to extend into the center grove of a wire bobbin when it is mounted on the rotor bases for wire reloading. 5. With the wire-clip mechanism installed, slide the adjustment plate around until the motor axis of rotation (dotted black line) is precisely in line with the wire bundle (red lines). When properly aligned, the apex of the wire bundle will appear motionless during motor turning.

The mechanical portion of Twister3 consists of common hardware, standard optomechanical components, and 3D-printed parts. Wherever possible, we used standard (and easy to replace) parts. The mechanical bill of materials is shown in table B1. In addition to these standard mechanical components, several 3D-printed parts are required (table B2). These parts are available for direct purchase from third-party 3D-printing services via the links provide in the table. After obtaining the required components, the wire feeder, wire-guide, and stock-spool assemblies can be constructed by following steps detailed figures 6 and 7(A)–(C), respectively. After each of these modules is complete, they are combined into the complete device by following the steps presented in figure 8.

### 4.2. Control electronics

The control board is comprised of the following blocks: power regulation, motor-driver, microcontroller, and user interface (figure 2(A)). Wherever possible, we used pre-assembled modules (microcontroller, motor driver, and LCD display). The bill of materials for this board is shown in table B3. Printed circuit board designs and Gerber files are available on the Twister3 repository^12^.

## 5. Discussion

There are several device options for making TWPs. These broadly fall into two categories: *(1)* Twisters with a manual wire folding step, and *(2)* Pre-loaded bobbin designs. Devices such as the Open Ephys Twister^13^, Matt Gaidica’s Simple Twister^14^, and the Neuralynx Spinner-2.0^15^ fall into the first category. The first of these two devices are very cheap and simple, and may be ideal for labs who do not need to make many TWPs and can accept some TWP construction variability. However, a general disadvantage with manual folding machines is that they are slow. This is due to a manual folding step combined with loose mechanical coupling that requires slow turning speeds. A single TWP generally takes several minutes to make, even for an experienced operator. This can be partially mitigated by using these devices in parallel: a second TWP is folded while the first is turned. However, we have found that due to the finicky nature of the folding step, using any more than two devices at a time is nearly impossible. Further it places a large rote labor burden on the operator, which can lead to poor construction quality due to boredom.

Aside from slow construction speed, these devices introduce large (and uncontrollable) variability in manual wire handling and twist-pitch (which is directly linked to electrode compliance (figure 4)). Although they are cheaper than bobbin-based designs, their manual labor requirements and slow operation lead to human-resource requirements that can far outweigh the increased material cost of bobbin-based designs.

There are two options for pre-loaded bobbin TWP machines: Twister3 and the SpikeGadgets Tetrode Machine^16^. Although similar in principle of operation, these two designs use different strategies at nearly every component resulting in very different user experiences and cost. We have summarized these differences in table 2.

The two most notable differences between Twister3 and the SpikeGadgets Tetrode Machine are the means by which they increase TWP construction speed and the wire fusing mechanism. Twister3 provides automatic wire bundle alignment and a leaf-spring based bundle to motor coupling, instead of relying on gravity to provide wire tension. This means that wire can be turned at fast speeds while maintaining twist integrity. Because wire turning only takes a few seconds, it is not a rate limiting step in the TWP construction process. Because the SpikeGadgets device lacks Twister3’s motor coupling features, it must turn TWPs relatively slowly (~60 RPM vs. ~1000 RPM).

To compensate for its slow turn rate, the SpikeGadgets machine permits efficient construction via parallelization. Wire is turned slowly, but a linear actuator and hot air gun are used to perform the fusing step automatically. The benefit of this strategy is twofold: (1) increased repeatability of the fusing step, and (2) manual labor is only required for clipping the wire bundle to the motor. This allows 3 identical twisting units to be used in parallel, increasing the effective TWP construction rate to the point where there is effectively no user downtime. The downside of this strategy is a major increase in materials cost and design complexity compared to Twister3. However, given the additional benefits of automated wire fusing, we see this feature as obvious target for future improvement of Twister3.

Aside from these two primary differences, Twister3 also affords several other improvements compared to the SpikeGadgets device. Twister3 provides precise acceleration control, quick-draw wire feeding, rapid magnetic wire bundle to motor attachment mechanism, and automatic wire tensioning. These features simplify operator use in comparison to the SpikeGadgets machine. Further, our use of standard optomechanical and cheap 3D printed parts greatly reduces BOM cost and increases ease of acquisition compared to SpikeGadgets design, which relies on custom, tight-tolerance machined parts.

A construction step that is not currently addressed by any TWP machine is the precision cut used to create the exposed probe tip. The most common ”final cut” method is to place the TWP in a single groove between the serrations of well-maintained, hard (tungsten or carbide-coated) scissors and then manually close them [15, 17]. This typically creates TWPs without electrical shorts, but does not result in consistent cross-sectional electrode geometry when the tips are viewed using electron microscopy [17]. More consistent geometry can be obtained by performing this cut with the shaft submerged in liquid nitrogen which makes the insulating material brittle. However, both of these procedures are tedious and variable and therefore lend themselves to automation. Automated TWP shearing could perhaps be incorporated into the Twister3 design in the future.

Because TWPs provide a good balance of data quality, ease of use, ease of assembly, and low cost, they will continue to be used in *in vivo* neurophysiology labs for years to come. Twister3 is a simple device that greatly decreases manual labor, greatly increases TWP production speed and quality, and is affordable for most labs that might benefit from it. It is fully open-source, well-documented (via this manuscript and instructional videos (appendix A)), and is composed of easy to obtain parts. Further, we encourage the replication and improvement of this device by others. For instance, an open design that incorporates the automated wire fusing step would further reduce human variability in TWP quality of the current design. We hope that Twister3 complements the growing number of open-source electrophysioloy tools, such as microdrives [5, 16, 6], electrical stimulators [18], optical stimulators [19]^17^, acquisition hardware [20], electrode impedance testers [21], and software [20, 22, 23]. Combined with a growing ecosystem of open-source hardware, e.g. for microscopic imaging [24–26], DNA amplification [27], audio monitoring [28], culture plate-reading [29], bacterial evolution [30], and closed-loop small animal experimentation [31, 32], these tools will permit labs to be completely outfitted with high-performance, low-cost, open-source tools. We believe this trend will increase the accessibility, transparency, and quality of scientific research in general [33].

## Figures and Tables

**Table 1.** Materials, operation parameters, and resulting tetrode features as Twister3 is used in our labs. Critical settings are the feeder height, which controls the TWP pitch and bobbin torsion, which controls wire tension.

Materials	Microwire	Mouse		Rat
		Sandvik PX000004		
**Hardware**	Feeder height		49 cm from base to bobbin axle	
**Settings**	Bobbin stiction		Set point 2 (~11.5 mN)	
	Hot air temp		480 °C	
	Hot air flow^a^		~5.8 L/m	
**Turn**	Forward turns	40		60
**Settings**	Reverse turns	10		15
	Turn speed		900 RPM	
**Results**	TWP length	37 mm		55 mm
	Twist pitch		1.18 mm	
	Avg. load time^b^		21.1 ± 10.0 sec	
	Turn time	8 sec		9.5 sec
	Avg. fuse time^b^		11.1 ± 5.6 sec	
	Avg. cut/removal time^b^		11.3 ± 4.5 sec	
	Avg. TWP time^b^	51.5 ± 9.6 sec		53.0 ± 9.6 sec

a Using the hot air station specified in the bill of materials, this is the lowest setting used in combination with 8 mm diameter nozzle

b Mean ± standard deviation over three novice users (figure 5)

**Table 2.** Comparison of Twister3 and SpikeGadgets Tetrode Machine.

	SpikeGadgets Tetrode Machine	Twister3 (this device)
Wire feed mechanism	Spring-loaded clamp	Torsion-spring quick draw
Automatic wire fusing	Yes	No
Wire/motor-axis alignment	No	Yes
TWP turning motor type	Continuous DC	Stepper
Wire fuser motor type	Stepper	N/A
Motor controller	Standard half-bridge	TMC2130
TWP turning speed	60 RPM typical	700–1000 RPM typical
Acceleration control	No	Yes
Bobbin/motor attachment	Screw	Magnets
Wire bundle/motor attachment	Free-hanging	Leaf-spring & magnet
Microcontroller module	Arduino Due	Teensy LC
Adjustable twist geometry	No	Yes
Mechanical parts	Custom machined	Standard optomechanics & 3D printed

## Footnotes

1 e.g. http://neuronexus.com/products/neural-probes/.

2 http://www.open-ephys.org/twister.

3 https://neuralynx.com/hardware/tetrode-spinner-2.0.

4 http://www.spikegadgets.com/main/home.html; Note: M. K. is co-owner and M. B. is an employee.

5 http://www.spikegadgets.com/hardware/tetmachine.html.

6 https://github.com/jonnew/twister3.

7 https://neuralynx.com/hardware/tetrode-spinner-2.0.

8 Polyimide-coated nichrome; ∅12.7*μ*m.

9 https://www.pjrc.com/teensy/teensyLC.html.

10 https://github.com/luni64/TeensyStep.

11 https://bit.ly/2H2a4FD.

12 https://github.com/jonnew/twister3/tree/master/control-board.

13 http://www.open-ephys.org/twister.

14 http://www.open-ephys.org/simple-twister.

15 https://neuralynx.com/hardware/tetrode-spinner-2.0.

16 http://www.spikegadgets.com/hardware/tetmachine.html.

17 https://github.com/jonnew/cyclops.

## Appendix A Instructional videos

Videos documenting device operation and usage are available on YouTube:

- https://youtu.be/xQbXc738ZuM Annotated video showing how to use Twister3 to load bobbins with electrode wire and how to use Twister3 to make tetrodes.
- https://youtu.be/B0MdM4z-wl0 Video showing the construction of two tetrodes, start to finish.

## Appendix B Bills of materials

**Table B1.** Mechanical bill of materials. A continuously updated bill of materials is located on this Google sheet.

Mechanical Bill of Materials			
Qty	Description	Supplier	Supplier Part No.
1	NEMA-17 Stepper Motor	Digikey	1460-1163-ND
10	3 × 7 × 3 mm Bearings	Boca Bearing	MR73-2RSC
1	M3 × 4 mm Set screw	McMaster-Carr	92605A098
2	M3 × 6 mm Standoff	McMaster-Carr	94868A162
1	M3 × 0.5 mm Thread Size, 500 mm Long	McMaster-Carr	90024A219
5	M3 × 0.5 mm Thread, 40 mm Long	McMaster-Carr	91292A024
2	M3 × 0.5 mm Thread, 60 mm Long	McMaster-Carr	92290A131
8	M6 × 1 mm Thread, 14 mm Long	McMaster-Carr	92855A615
2	M6 × 1 mm Thread, 25 mm Long, Set screw	McMaster-Carr	92015A135
4	Music-Wire Steel Torsion Spring	McMaster-Carr	9271K578
2	Zinc-Plated, M3 × 0.5 mm Thread Nut	McMaster-Carr	90695A033
2	Dowel Pin 3/16” Diameter, 2-1/2” Long	McMaster-Carr	98380A520
2	∅ 1.5” Mounting Post, M6 Taps, L = 250 mm	ThorLabs	P250/M
2	Swivel-Base Mini-Post Holder, 2” (51 mm) Tall, 1/4” (M6) Slot	ThorLabs	MSL2
2	Mini-Series Optical Post, ∅ 6 mm, L = 50 mm	ThorLabs	MS2R/M
1	Aluminum Breadboard, 300 mm × 600 mm × 12.7 mm, M6 Taps	ThorLabs	MB3060/M
1	∅ 1.5” Post Mounting Clamp, 112.5 mm × 112.5 mm, Metric	ThorLabs	C1545/M
10	Neodymium Block Magnets, 1/8” cube	KJ Magnetics	B222G-N52
2	Neodymium Block Magnets, 1/2” × 1/4” × 1/16”	KJ Magnetics	B841-N52
2	Wire Clip	Digikey	314–1017-ND
NA	Silicone Heat Shrink	Digikey	XX

**Table B2.** 3D-printing bill of materials. An continuously updated bill of materials is located on this Google sheet. The right-most column provides links to order parts from each supplier.

3D-Printing Bill of Materials					
Qty	Description	Material	Supplier	STL File in Repository	Link
1	Feeder Base	Nylon PA12	Shapeways	twister3_arm-base.STL	http:/shpws.me/QtfT
1	Motor Mount	Nylon PA12	Shapeways	twister3_motor-mount.STL	http:/shpws.me/QtfL
1	Bobbin Shield	‘Fine detail plastic’	Shapeways	twister3_bobbin-shield.STL	http:/shpws.me/QtfU
2	Bobbin Spacer	‘Fine detail plastic’	Shapeways	twister3_bobbin-spacer.STL	http:/shpws.me/QtfR
2	Bobbin Washer	‘Fine detail plastic’	Shapeways	twister3_bobbin-washer.STL	http:/shpws.me/QtfP
4	Bobbin	‘Fine detail plastic’	Shapeways	twister3_bobin.STL	http:/shpws.me/QtfM
1	Rotor Base	‘Fine detail plastic’	Shapeways	twister3_rotor-base.STL	http:/shpws.me/QtfK
1	Adjustment Plate	‘Fine detail plastic’	Shapeways	twister3_adjustment-plate.STL	http://shpws.me/Rekt
1	Wire Guide	‘Fine detail plastic’	Shapeways	twister3_wire-guide-base.STL	http:/shpws.me/QtfI
2	Stock Spool Bearing Case	‘Fine detail plastic’	Shapeways	twister3_stock-spool-bearing-case.STL	http:/shpws.me/QUtz
1	Twist Wire Aligner	Rhodium plated brass	Shapeways	twister3_twist-aligner.STL	http://shpws.me/R6mo
1	Spring Rotor Top	PEBA 2301	Sculpteo	twister3_spring-top.STL	https://bit.ly/2H32cna

**Table B3.** Control box (figure 2) bill of materials. A continuously updated bill of materials is located on this Google sheet. Table entries labeled ‘common’ are ubiquitous and therefore listing specific part numbers is futile due to rapid supplier turn over. PCB design files are located at this link. The PCB is split into two halves separated by a breakaway v-cut. One half serves as the top panel of the enclosure, visible in (figure 2(B)), and the other has all electronics and is mounted beneath the front panel.

Control Box Bill of Materials						
Qty	Schematic Name	Schematic Value	Description	Supplier	Supplier Part No.	Manufacturer Part No.
1	U5	N/A	Teensy 3.2	PRJC		
3	M1, M2, M3	N/A	M3 Screws, 10 mm	McMaster	92000A120	
1	N/A	N/A	24 V Wall Adapter	Amazon		
1	S1	EC12P	Rotary Encoder	Sparkfun	10982	EC12P
1	S1	N/A	Knob	Sparkfun	10597	P-15×13-T-T2-24A
1	U6	GDM1602K	LCD Display 5 V	Sparkfun	709	GDM1602K
1	N/A	N/A	Hot-Air Station	Sparkfun	14557	
1	For U5	Teensy Header	2×7 Header	Digikey	609-3485-1-ND	61001421121
2	For U5	Teensy Header	1×14 Header	Digikey	732-5325-ND	61301411121
1	For U5	Teensy Socket	2×7 Socket	Digikey	S7110-ND	PPPC072LFBN-RC
2	For U5	Teensy Socket	1×14 Socket	Digikey	S7012-ND	PPTC141LFBN-RC
1	For U6	LCD Header	1×16 Header	Digikey	732-5327-ND	61301611121
1	For U6	LCD Socket	1×16 Socket	Digikey	S7014-ND	PPTC161LFBN-RC
2	J10, J11	Stepper Stick Header	1×8 Header	Digikey	732-5321-ND	61300811121
2	J10, J11	Stepper Stick Socket	1×8 Socket	Digikey	S7006-ND	PPTC081LFBN-RC
5	J1, J8, J9	N/A	1x4 Socket	Digikey	S7002-ND	PPTC041LFBN-RC
3	J3, J4, J5	N/A	1x4 SMD Header	Digikey	SAM12225-ND	TSM-104-02-L-SV
2	For U2	N/A	Board Spacers	Digikey	952-3465-ND	R6010-00
3	M4, M5, M6	N/A	M3 SMD Nuts	Digikey	732-10918-1-ND	9775056360 R
1	J2	N/A	Screw Terminal	Digikey	ED10563-ND	OSTVN04A150
1	J1	PJ-063BH	Barrel Jack	Digikey	CP-063BH-ND	PJ-063BH
4	C2, C6, C7, C8	10 nF, 50 V	Capacitor, X7R	Digikey	478-1227-1-ND	06035C103KAT2A
1	C16	470 nF, 10 V	Capacitor, X7R	Digikey	1763-1190-1-ND	0402BW474K160YT
2	C1, C3	1 uF, 50 V	Capacitor, X7R	Digikey	587-3247-1-ND	UMK107AB7105KA-T
1	C4	15 uF	Tantalum Capacitor	Digikey	399-3775-1-ND	T491D156K035AT
1	C5	220 uF	Tantalum Capacitor	Digikey	399-9744-1-ND	T491D227M010AT
1	R6	470 Ω	Resistor, Thick film	Digikey	Common	Common
2	R1, R9, R10, R11, R12	4.7 kΩ	Resistor, Thick film	Digikey	Common	Common
5	R2, R3, R4, R5, R17	10 kΩ	Resistor, Thick film	Digikey	Common	Common
1	R13	20 kΩ	Resistor, Thick film	Digikey	Common	Common
2	R8, R16	20 kΩ	Trim Pot	Digikey	SM-42TX203CT-ND	SM-42TX203
1	L1	68 uH	Inductor	Digikey	513-1386-1-ND	DR125-680-R
1	D1	DB2W60400L	Schottky Diode	Digikey	P15184CT-ND	DB2W60400L
1	U1	IS31FL3193	LED Driver	Digikey	706-1215-1-ND	IS31FL3193-DLS2-TR
1	U3	MAX6816	Debouncer	Digikey	MAX6816EUS+TCT-ND	MAX6816EUS+T
1	U2	MIC4680-5.0YM-TR	DC DC Converter	Digikey	576-1221-1-ND	MIC4680-5.0YM-TR
1	U4	TMC2100	Silent Stepper Stick	Digikey	1460-1159-ND	N/A
1	N/A	N/A	Enclosure	Digikey	36-705-ND	705
